# IFN-γ protects from lethal IL-17 mediated viral encephalomyelitis independent of neutrophils

**DOI:** 10.1186/1742-2094-9-104

**Published:** 2012-05-29

**Authors:** Carine Savarin, Stephen A Stohlman, David R Hinton, Richard M Ransohoff, Daniel J Cua, Cornelia C Bergmann

**Affiliations:** 1 Department of Neurosciences NC30, Lerner Research Institute, The Cleveland Clinic Foundation, 9500 Euclid Avenue, Cleveland, OH, 44195, USA; 2 Department of Pathology, Keck School of Medicine, University of Southern California, 2011 Zonal Avenue, Los Angeles, CA, 90033, USA; 3 Merck Research Laboratories, DNAX Discovery Research, 901 California Ave, Palo Alto, CA, 94304, USA

**Keywords:** Central nervous system, Encephalomyelitis, CD4^+^ T cells, IFN-γ, IL-17, Neutrophils, Neurotropic coronavirus

## Abstract

**Background:**

The interplay between IFN-γ, IL-17 and neutrophils during CNS inflammatory disease is complex due to cross-regulatory factors affecting both positive and negative feedback loops. These interactions have hindered the ability to distinguish the relative contributions of neutrophils, Th1 and Th17 cell-derived effector molecules from secondary mediators to tissue damage and morbidity.

**Methods:**

Encephalitis induced by a gliatropic murine coronavirus was used as a model to assess the direct contributions of neutrophils, IFN-γ and IL-17 to virus-induced mortality. CNS inflammatory conditions were selectively manipulated by adoptive transfer of virus-primed wild-type (WT) or IFN-γ deficient (GKO) memory CD4^+^ T cells into infected SCID mice, coupled with antibody-mediated neutrophil depletion and cytokine blockade.

**Results:**

Transfer of GKO memory CD4^+^ T cells into infected SCID mice induced rapid mortality compared to recipients of WT memory CD4^+^ T cells, despite similar virus control and demyelination. In contrast to recipients of WT CD4^+^ T cells, extensive neutrophil infiltration and IL-17 expression within the CNS in recipients of GKO CD4^+^ T cells provided a model to directly assess their contribution(s) to disease. Recipients of WT CD4^+^ T cells depleted of IFN-γ did not express IL-17 and were spared from mortality despite abundant CNS neutrophil infiltration, indicating that mortality was not mediated by excessive CNS neutrophil accumulation. By contrast, IL-17 depletion rescued recipients of GKO CD4^+^ T cells from rapid mortality without diminishing neutrophils or reducing GM-CSF, associated with pathogenic Th17 cells in CNS autoimmune models. Furthermore, co-transfer of WT and GKO CD4^+^ T cells prolonged survival in an IFN-γ dependent manner, although IL-17 transcription was not reduced.

**Conclusions:**

These data demonstrate that IL-17 mediates detrimental clinical consequences in an IFN-γ-deprived environment, independent of extensive neutrophil accumulation or GM-CSF upregulation. The results also suggest that IFN-γ overrides the detrimental IL-17 effector responses via a mechanism downstream of transcriptional regulation.

## Background

IL-17 and IFN-γ play diverse and often opposing functions during microbial infections, as well as autoimmune diseases. These interactions are partially attributed to their distinct regulation of the neutrophil response. Both IL-17A and IL-17 F signal through the IL-17R to induce granulocyte colony-stimulating factor and stem cell factor, thereby expanding neutrophil progenitors in the bone marrow and spleen as well as increasing mature neutrophils in the blood [1-3]. IL-17 also induces ELR^+^ CXC chemokines, which attract neutrophils [2,3]. By contrast, IFN-γ opposes neutrophil recruitment by downregulating expression of neutrophil chemoattractants [4]. Analysis of polarized T cell subsets and genetically deficient mice has provided insight into the distinct effector functions of IL-17 and IFN-γ; however, the interplay between IL-17 and IFN-γ *in vivo* remains complex [5,6]. Moreover, downstream effector mechanisms mediating pathological consequences may be tissue- and pathogen-specific and are largely unresolved. For example, Th17 cell-mediated protection is critical during bacterial pneumonia [2]. IL-17-mediated neutrophil recruitment to the infection site also indicates a protective role for Th17 cells during oropharyngeal candidiasis [7]. By contrast, Th17-mediated inhibition of both protective Th1 responses and antimicrobial neutrophil functions increased tissue destruction following gastric candidiasis and pulmonary aspergillosis [8]. These differences may reflect distinct infection sites, as indicated by the distinct immune responses to *Candida albicans*, which are dependent upon the anatomical site of infection [7].

Viral infections are often dominated by Th1 responses. However, the coemergence of Th17 and Th1 cells has recently been documented in several infections, including human immunodeficiency virus [9], simian immunodeficiency virus [10] and cytomegalovirus [11]. A deleterious role of IL-17 is implied by acute lung injury associated with IL-17-mediated neutrophil recruitment during influenza virus infection [12]. By contrast, Th17 responses are protective against lethal influenza virus infection in IL-10-deficient mice [13]. Similarly, IFN-γ-mediated protection during herpes simplex virus-1 corneal infection correlated with reduced IL-17 production and subsequent neutrophil infiltration [14]. However, the function of IL-17 during central nervous system (CNS) viral infections, including human immunodeficiency virus encephalitis, is unclear, although Th17 cells promote Theiler’s murine encephalomyelitis virus persistence and chronic demyelination by limiting the antiviral cytotoxic T-lymphocyte response [15].

In contrast to the limited information on IL-17 function during viral encephalitis, analysis of experimental autoimmune encephalitis (EAE) has revealed numerous insights into effector mechanisms as well as crosstalk between Th1 and Th17 cells [16]. Although the inflammatory CNS disease multiple sclerosis and its animal model EAE were historically associated with a Th1 immune response [17,18], a pro-inflammatory role of IFN-γ was contradicted by substantially increased disease severity and mortality in mice deficient in IFN-γ (GKO) or the IFN-γR [19,20]. The correlation between increased EAE severity, enhanced Th17 responses and neutrophil infiltration into the CNS of GKO mice suggested that IFN-γ might be protective by inhibiting the Th17 response [21]. Although IL-17^−/−^ mice are susceptible to EAE [22], adoptive transfer of polarized encephalitogenic CD4^+^ T cells support Th17 cells as detrimental participants in EAE [23,24]. However, the pathogenic mechanisms associated with Th17 cells remain an ongoing challenge and may involve multiple pathways. These include excessive CNS neutrophil infiltration and release of degrading enzymes, free radicals and pro-inflammatory cytokines, direct IL-17-mediated neuronal toxicity [25], and/or secretion of granulocyte macrophage colony-stimulating factor (GM-CSF) as the pathogenic effector molecule [26-28]. These data suggest that the balance between IFN-γ and IL-17 effector functions, as well as their regulation of neutrophils may dictate the outcome of non autoimmune-driven CNS inflammation, such as viral encephalitis.

During encephalomyelitis induced by the strain designated JHMV, CD4^+^ T cells not only contribute to antiviral effects by enhancing CD8^+^ T cell function within the CNS [29] but also mediate viral control in absence of CD8^+^ T cells [30]. Nevertheless, they also contribute to both clinical disease and demyelination [30]. To define the role of CD4^+^ relative to CD8^+^ T cells in viral encephalitis, memory CD4^+^ T cells from immunized donors were transferred into infected severe combined immunodeficiency (SCID) mice [31]. This study revealed an early morbidity and mortality in infected recipients of CD4^+^ T cells lacking the ability to secrete IFN-γ compared to recipients of IFN-γ-sufficient CD4^+^ T cells or infected unreconstituted control mice [31]. Notably, both memory populations were equally effective in controlling virus replication [31]. The lethal outcome was specific for CD4^+^ T cells lacking IFN-γ [31], but not for a similar memory CD8^+^ T cell population deficient in IFN-γ [32]. These data suggest that mortality was related to immune effector functions specific to CD4^+^ T cells and controlled by IFN-γ.

In this study, SCID recipients of GKO CD4^+^ T cells infected with JHMV were characterized by extensive neutrophil accumulation and IL-17 expression within the CNS. Neutrophil infiltration in the absence of IFN-γ correlated with significantly elevated levels of CXCL1, independent of IL-17. Moreover, comparison of infected recipients of wild-type (WT) CD4^+^ T cells depleted of IFN-γ and recipients of GKO CD4^+^ T cells depleted of IL-17 revealed mortality was due to IL-17, irrespective of abundant neutrophil accumulation. IFN-γ introduced by co-transfer of WT CD4^+^ T cells with IL-17-producing GKO CD4^+^ T cells abrogated the detrimental effects of IL-17 without affecting IL-17 transcription within the CNS. These data thus segregate the effects of toxic neutrophil components from IL-17-mediated pathogenesis.

## Material and Methods

### Mice

Homozygous BALB/c Thy1.1 mice, provided by Dr. J. Harty (University of Iowa, Iowa City, IA, USA) and GKO BALB/c mice, provided by Dr. R. Coffman (DNAX Research, Palo Alto, CA, USA), were bred locally at the Cleveland Clinic. SCID mice were obtained from the National Cancer Institute (Frederick, MD, USA). Recipients and donors were maintained under sterile conditions and all procedures were performed in compliance with Cleveland Clinic Institutional Animal Care and Use Committee-approved protocols.

### Virus

The gliatropic JHM strain of mouse hepatitis virus (JHMV)-neutralizing mAb variant designated 2.2v-1 was used for intracerebral infection [33]. JHMV was propagated and plaque assayed on monolayers of DBT cells, a continuous murine astrocytoma cell line [32]. SCID mice were injected in the left hemisphere with 30 μl volume containing 500 PFU of JHMV diluted in endotoxin-free Dulbecco’s modified PBS. The severity of the JHMV-induced clinical disease was graded as follows: 0, healthy; 1, ruffled fur and hunched back; 2, partial hind limb paralysis or inability to turn to the upright position; 3, complete hind limb paralysis; 4, moribund or dead. Virus titers were determined on plaque assay on monolayers of DBT cells as previously described [32,33]. Briefly, brains were homogenized in ice-cold Dulbecco’s PBS using Ten Broeck tissue homogenizers (Kimble Chase, Vineland, NJ, USA). After clarification by centrifugation at 400 x g for 7 minutes at 4°C, supernatants were stored at −70°C whereas pellets containing CNS-derived cells were suspended in Percoll (GE Healthcare Bio-Sciences AB, Uppsala, Sweden) and used for flow cytometry analysis (see below).

### T cell purification and adoptive transfer

BALB/c Thy1.1 and GKO donors were immunized by intraperitoneal (i.p.) injection with 2 × 10^6^ PFU of JHMV. Donor splenocytes were prepared four to sixteen weeks post immunization. CD4^+^ T cells were purified by positive selection using anti-CD4-coated magnetic beads (Miltenyi Biotec Inc., Auburn, CA, USA). Purity of the purified population was assessed by flow cytometry using fluorescein isothiocyanate- (FITC) labeled anti-CD4 (clone GK1.5), phycoerythrin- (PE) labeled anti-CD8 (clone 53-6.7) and peridinin chlorophyll protein- (PerCP) labeled anti-CD19 (clone 1D3) mAbs (BD Pharmingen, San Diego, CA, USA). Recipients received 5 × 10^6^ donor CD4^+^ T cells composed of 100% Thy1.1 (WT), 100% GKO or a 50/50% mixture of Thy1.1/GKO (WT/GKO) CD4^+^ T cells by intravenous (i.v.) injection coupled with a single i.p. injection of 250 μg of anti-CD8 mAb (clone TIB.210). Mice were challenged with virus two to three hours after adoptive transfer. For neutrophil depletion, mice received i.p. injections of either 500 μg of anti-Ly-6G (clone 1A8) or anti-Gr1 (clone RB6-8C5) mAb every other day until sacrifice, starting two days before infection. Depletion was confirmed in both cases by flow cytometric analysis using anti-Ly-6G (clone 1A8) mAb in addition to examination of hematoxylin and eosin- (H&E) stained sections of brain. Only data for the anti-Ly6G experiments are shown. No differences in survival relative to control-treated mice were observed following treatment with either neutrophil-depleting mAb. Similarly, for anti-IFN-γ treatment, mice received i.p. injections of 500 μg of anti-IFN-γ (clone XMG1.2) mAb every other day, starting two days before infection. For anti-IL17 treatment, mice received i.p. injections of 1 mg of anti-IL-17A (clone 1D10) mAb at day zero and six post infection (p.i.).

### Isolation of central nervous system-derived cells

After brain homogenization and centrifugation to obtain supernatants for virus determination as described above, cell pellets were resuspended in RPMI containing 25 mM HEPES, pH 7.2 and adjusted to 30% Percoll (GE Healthcare Bio-Sciences BA). A 1 ml underlay of 70% Percoll was added prior to centrifugation at 800 x g for 30 minutes at 4°C. Cells were recovered from the 30% to 70% interface and washed with RPMI medium prior to analysis.

### Flow cytometry

CNS mononuclear cell suspensions were blocked with anti-mouse CD16/CD32 (clone 2.4G2, BD Pharmingen) mAb on ice for 15 minutes prior to staining. Cells were then stained with FITC-, PE-, PerCP- or allophycocyanin-conjugated mAb for 30 minutes on ice in PBS containing 0.1% BSA. Expression of surface molecules was characterized using the following mAbs (all obtained from BD Pharmingen except when indicated): anti-CD45 (Clone Ly-5), anti-CD4 (clone GK1.5), anti-Thy1.1 (clone OX-7), anti-CD8 (clone 53-6.7), anti-CD11b (clone M1/70), anti-F4/80 (Serotec, Oxford, UK), anti-Ly6G (clone 1A8) and anti I-A/I-E (clone 2G9). Samples were analyzed on a FACSCalibur flow cytometer using CellQuest software (Becton Dickinson, Mountain View, CA, USA).

### Gene expression analysis

RNA was isolated from three or more individual brains per group using TRIzoL reagent (Invitrogen, Carlsbad, CA, USA) according to the manufacturer’s instructions. cDNAs were prepared using SuperScript II Reverse Transcriptase (Invitrogen) and oligo (dT)_12–18_ primers (Invitrogen). Semi-quantitative RNA expression was assessed using LightCycler and SYBR Green kit (Roche, Basel, Switzerland) and the following primers; ubiquitin: F: 5’- TGGCTATTAATTATTCGGTCTGCAT-3’, R: 5’- GCAAGTGGCTAGAGTGCAGAGTAA -3’; IFN-γ: F: 5’- TGATGGCCTGATTGTCTTTCAA-3’, R: 5’- GGATATCTGGAGGAACTGGCAA-3’; IL-17: F: 5’-CTTCATCTGTGTCTCTGATGCTGTT-3’, R: 5’- TCGCTGCTGCCTTCACTGT-3’; IL-22: F: 5’- CATGCAGGAGGTGGTACCTT-3’, R: 5’- CAGACGCAAGCATTTCTCAG-3’; IL-21: F: 5’- GGACAGTATAGACGCTCACGAATG-3’, R: 5’- CGTATCGTACTTCTCCACTTGCA-3’; MHC class II: F: 5’- TCAACATCACATGGCTCAGAAATA-3’, R: 5’- AGACAGCTTGTGGAAGGAATGG-3’; GM-CSF: F: 5’- TTTCCTGGGCATTGTGGTCTA -3’, R: 5’- AAGGCCGGGTGACAGTGAT -3’; IL-6: F: 5’- ACACATGTTCTCTGGGAAATCGT -3’, R: 5’- AAGTGCATCATCGTTGTTCATACA-3’; IL-1β: F: 5’- GACGGCACACCCACCCT-3’, R: 5’- AAACCGTTTTTCCATCTTCTTCTTT-3’; CCL7: F: 5’-GGGAAGCTGTTATCTTCAAGACAAA-3’, R:5’-CTCCTCGACCCACTTCTGATG-3’; CCL20: F: 5’-GGTGGCAAGCGTCTGCTC-3’, R: 5’-GCCTGGCTGCAGAGGTGA-3’; CXCL2: F: 5’-CCTGCCAAGGGTTGACTTCA-3’, R: 5’-TTCTGTCTGGGCGCAGTG-3’; MMP9: F: 5’- CCATGCACTGGGCTTAGATCAT-3’, R: 5’- CAGATACTGGATGCCGTCTATGTC-3’; MMP3: F: 5’- TTTAAAGGAAATCAGTTCTGGGCTATA-3’, R: 5’-CGATCTTCTTCACGGTTGCA-3’; MMP12: F: 5’- GGAGCTCACGGAGACTTCAACT-3’, R: 5’-CCTTGAATACCAGGTCCAGGATA -3’. TaqMan primers and 2X TaqMan fast master mix (Applied Biosystems, Carlsbad, CA, USA) were used to assessed CXCL1 and CCL2 mRNA levels. Levels of mRNA expression were normalized to ubiquitin mRNA using ΔCt method as previously described [31].

### Immunofluorescence

After ice-cold PBS perfusion, brains in OCT were frozen in liquid nitrogen and stored at −80°C until 10 μm sections were prepared. Sections were fixed with methanol/acetone (1:1 ratio) for 15 minutes and then treated with blocking solution for 30 minutes at room temperature. Rat anti-mouse IL-17 (R&D systems, Minneapolis, MN, USA) and hamster anti-mouse CD3 primary mAbs (Serotec) were incubated overnight at 4°C. Alexa Fluor 488 goat anti-rat (Invitrogen) and Alexa Fluor 546 goat anti-hamster (Molecular Probes, Eugene, OR, USA) were added for 1 hour at room temperature. Sections were mounted with Vectashield mounting medium with 4’-6-Diamidino-2-phenylindole (DAPI) (Vector Laboratories, Burlingame, CA, USA) and analyzed using a Leica DM4000B fluorescent microscope (Leica, Wetzlar, Germany).

### In vitro T cell stimulation

Cytokine expression by CD4^+^ T cells derived from cervical lymph nodes of SCID recipients were analyzed directly at day eight p.i. without stimulation with viral antigen. For analysis of cytokine production by cells prior to transfer, JHMV was adsorbed to donor splenocytes for 60 minutes at 4°C and cells cultured for six days in RPMI complete, 10% FCS at 2.5 × 10^6^ cells/ml. Cytokine production from both splenic cultures or *ex vivo* lymph node cells was measured following four hours stimulation with PMA (10 ng/ml) (Acros Organics, Geel, Belgium) and ionomycin (1 μM) (Calbiotech, Spring Valley, CA, USA). Monensin (2 μM) (Calbiotech) was added to the cultures for the last two hours. After stimulation, cells were harvested and stained for surface expression of CD4. Cells were then permeabilized using the cytofix/cytoperm kit (BD Pharmingen) according to the manufacturer’s instructions and stained for intracellular FITC-IFN-γ and PE-IL-17.

### Statistical analyses

Statistical differences were calculated using the two-tailed unpaired Student’s *t*-test. *P* values <0.05 were considered significant. *p < 0.05, **p < 0.01, ***p < 0.001

## Results

### IFN-γ mediated control of central nervous system neutrophil infiltration is not the sole factor regulating survival

**Figure 1:**
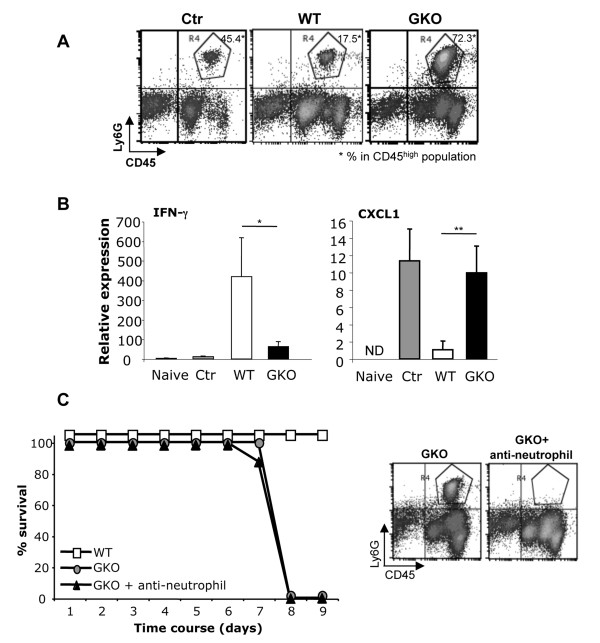
**Neutrophil depletion does not prevent early mortality. (A)** Neutrophil infiltration was characterized by flow cytometry based on CD45^hi^ Ly6G^+^ expression (R4 region) in the CNS of controls (control (ctr); infected SCID mice without CD4^+^ T cell transfer), infected SCID recipients of WT (WT) or GKO CD4^+^ T cells (GKO) eight days p.i. **(B)** Mean expression (±SEM) of IFN-γ and CXCL1 mRNA analyzed in brains of naïve (n = 4), controls (n = 3), SCID recipients of WT (n = 4) or GKO (n = 4) CD4^+^ T cells at day eight p.i. Data are representative of two experiments. **(C)** Survival rates of SCID recipients of WT or GKO CD4^+^ T cells (with and without neutrophil depletion) observed daily following JHMV infection. Data represent the mean of sixteen mice per group combined from two separate experiments with n = 8 per group in each experiment. Efficiency of neutrophil (CD45^hi^ Ly6G^+^, R4 region) depletion after antibody (Ab) treatment analyzed by flow cytometry in the CNS of SCID recipients of GKO CD4^+^ T cells at day eight p.i. Density plots are representative of eight animals per group.

One characteristic of GKO CD4^+^ T cell recipients infected with JHMV was the large CNS infiltrating neutrophil population (72.3% compared to 17.5% in WT CD4^+^ T cells recipients) (Figure 1A) [31]. Increased neutrophil accumulation in GKO recipients is consistent with IFN-γ-mediated downregulation of ELR^+^ neutrophil chemokines [4]. Indeed, analysis of cytokine and chemokine mRNA expression in infected T cell recipients demonstrated that high IFN-γ mRNA correlated inversely with mRNA expression of the neutrophil chemoattractant CXCL1 (Figure 1B). Thus, IFN-γ mRNA in WT CD4^+^ T cell recipients was associated with sparse CXCL1 expression and neutrophil recruitment, while low IFN-γ mRNA expression in both GKO CD4^+^ T cell recipients and infected SCID controls correlated with high CXCL1 expression and extensive neutrophil recruitment. Infected mice were depleted of neutrophils to explore a possible correlation between neutrophil-derived proteases, free radicals and proinflammatory cytokines with virus-induced mortality. Depletion was confirmed by the absence of Ly6G^+^ CD11b^+^ neutrophils within the CNS-derived inflammatory cells (Figure 1C). However, the absence of neutrophils did not prevent early mortality of GKO CD4^+^ T cell SCID recipients (Figure 1C), implicating alternate mechanisms inducing mortality in GKO recipients.

**Figure 2:**
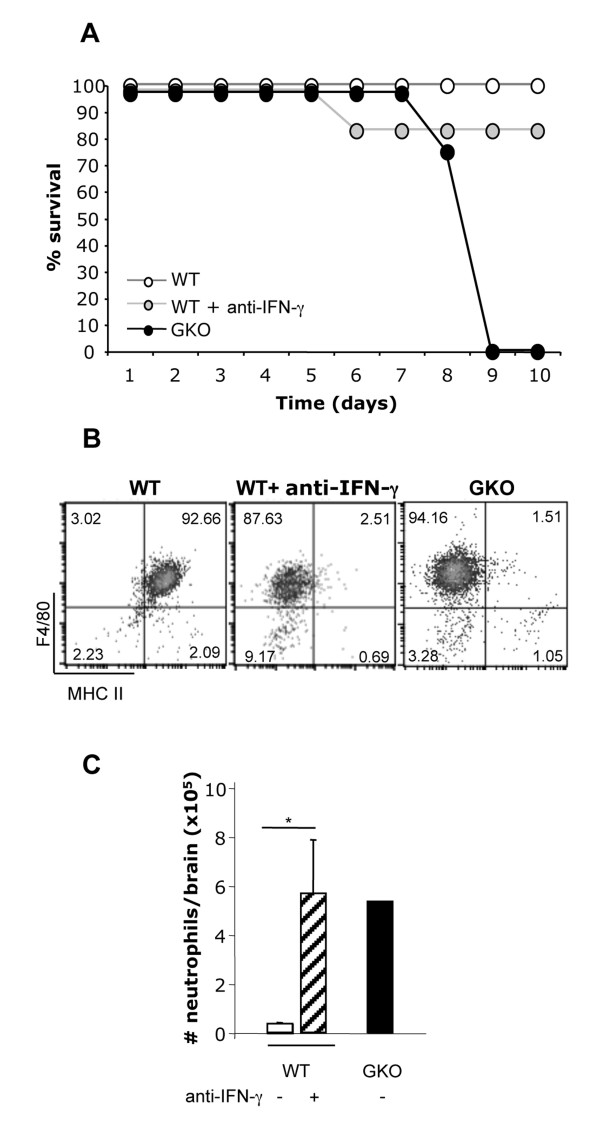
**Anti-IFN-γ induced CNS neutrophil infiltration but did not increase mortality in SCID recipients of WT CD4**^**+**^**T cells. (A)** Survival of infected SCID recipients of WT CD4^+^ T cells in the absence or presence of anti-IFN-γ mAb and recipients of GKO CD4^+^ T cells assessed daily until day ten p.i. Data are mean of at least six mice per group combined from two separate experiments **(B)** Anti-IFN-γ mAb efficiency assessed by flow cytometry by measuring MHC class II expression on microglia (CD45^lo^ F4/80^+^) in infected SCID recipients of WT (n = 6), WT + anti-IFN-γ mAb (n = 8) or GKO (n = 8) CD4^+^ T cells. Data are representative of two experiments. **(C)** Numbers of infiltrating neutrophils in the brain of infected SCID recipients of WT, WT + anti-IFN-γ mAb or GKO CD4^+^ T cells determined by flow cytometry at day eight p.i. Data represent the mean (±SD) from two independent experiments (n = 6, untreated group and n = 8, anti-IFN-γ treated group).

In contrast to memory GKO CD4^+^ T cells derived from JHMV-immunized donors, memory GKO CD8^+^ T cells did not trigger early mortality in infected SCID recipients [32]. These data suggest that IFN-γ deficiency was not the sole factor controlling early death. WT CD4^+^ T cell recipients were depleted of IFN-γ to confirm that a CD4^+^ T cell factor distinct from IFN-γ controls disease outcome. The modestly reduced survival rate of IFN-γ-depleted WT CD4^+^ T cell recipients (Figure 2A) demonstrated IFN-γ blockade did not reproduce the mortality of GKO CD4^+^ T cell recipients. The efficiency of IFN-γ blockade within the CNS was confirmed by analyzing IFN-γ-dependent MHC class II expression on microglia [34]. In contrast to class II expression on the vast majority of microglia in recipients of WT CD4^+^ T cells, class II remained undetectable in anti-IFN-γ-treated WT recipients (Figure 2B), confirming inhibition of local IFN-γ signaling within the CNS. IFN-γ depletion also had minimal effects on T cell recruitment into the CNS, reducing the CD4^+^ T cells within the inflammatory population from 15.4% to 12.3% (data not shown). In support of the role of IFN-γ in regulating neutrophils, IFN-γ-depleted WT recipients exhibited vastly increased CNS neutrophil infiltration, approaching the numbers found in GKO CD4^+^ T cell recipients (Figure 2C). In addition to confirming IFN-γ-mediated control of CNS neutrophil recruitment [4], these data reassert that abundant CNS neutrophils are insufficient to account for early mortality.

### IL-17 mediates mortality, independent of neutrophils

**Figure 3:**
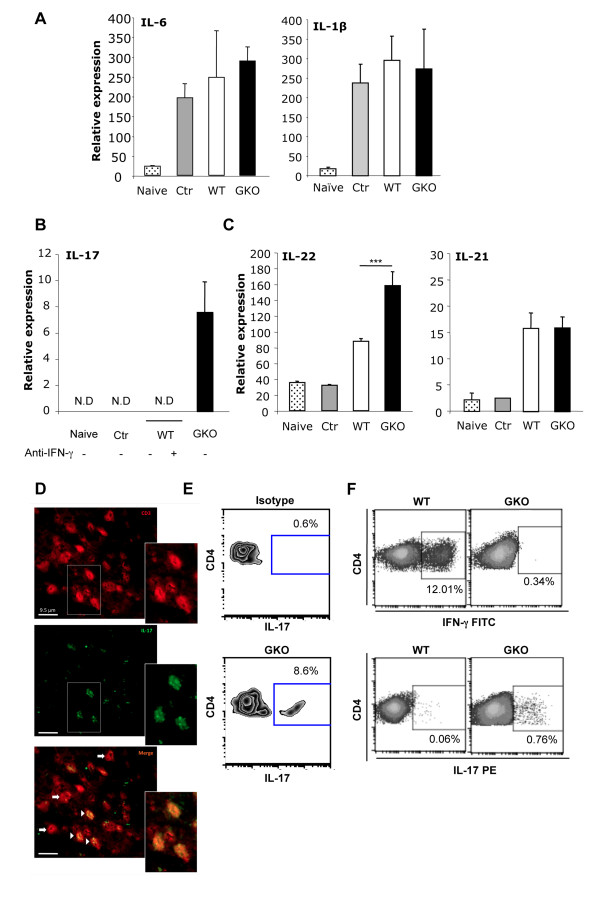
**IL-17 expression in SCID recipients of GKO CD4**^**+**^**T cells.** IL-6 and IL-1β **(A**), IL-17 **(B)**, and IL-22 and IL-21 **(C)** mRNA expression analyzed by quantitative real-time PCR in brains of naïve (n = 4), infected control (n = 3), infected SCID recipients of WT (n = 4) or GKO (n = 8) CD4^+^ T cells at day eight p.i. IL-17 mRNA expression was measured in infected SCID recipients of WT CD4^+^ T cells + anti-IFN-γ mAb (n = 8). Data represent the mean (±SEM) from two separate experiments. **(D)** IL-17 (green) and CD3 (red) expression in the brains of SCID recipients of GKO CD4^+^ T cells. CD3^+^ (arrows) and IL-17-producing T cells (arrow heads) detected at day eight p.i. **(E)** IL-17-expressing CD4^+^ T cells from cervical lymph nodes of SCID recipients of GKO CD4^+^ T cells at day eight p.i. Dot plots are representative of four individuals. **(F)** Expression of IFN-γ and IL-17 by donor-derived CD4^+^ T cells prior to transfer analyzed by flow cytometry after *in vitro* stimulation. Intracellular cytokine expression measured using FITC-IFN-γ, PE-IL-17 and the corresponding isotype controls. Dot plots are representative of duplicates from two separate experiments. N.D= Not Detected.

**Figure 4:**
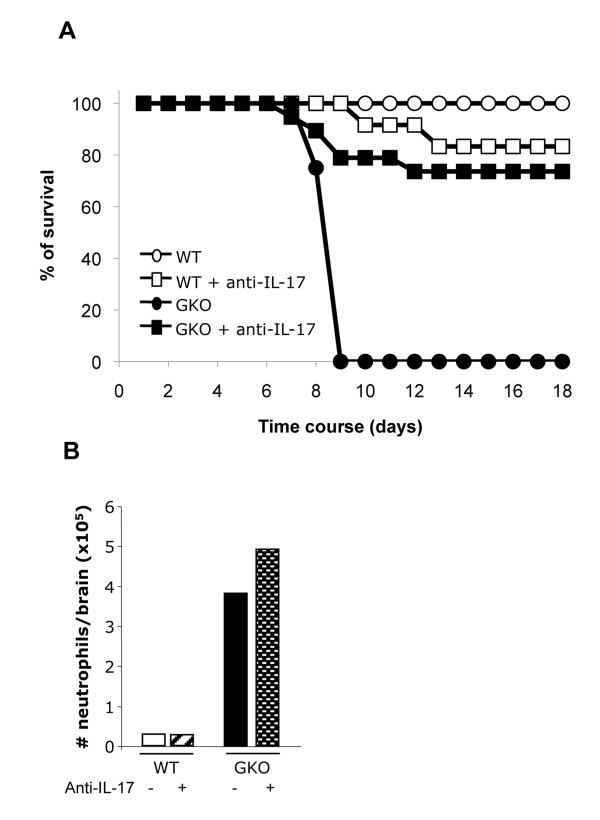
**IL-17 mediates mortality, independent of CNS neutrophil infiltration. (A)** Survival was assessed daily in infected SCID recipients of WT (n = 8), WT + anti-IL-17 mAb (n = 12), GKO (n = 8) and GKO + anti-IL-17 mAb (n = 12) CD4^+^ T cells until day 18 p.i. Data are mean from two separate experiments **(B)** Total numbers of neutrophils determined by flow cytometry at day eight p.i. in infected SCID recipients of WT, WT + anti-IL-17 mAb, GKO, GKO + anti-IL-17 mAb CD4^+^ T cells. Data represent the mean from two independent experiments (n = 4, untreated mice and n = 8, anti-IL-17-treated mice per experiment).

Neither IL-6 nor IL-1β, whose over expression is associated with adverse effects on the CNS [35,36], were increased in the CNS of GKO compared to WT CD4^+^ T cell recipients (Figure 3A). Previous data demonstrated that TNF and inducible nitric oxide synthase were also not associated with early mortality of GKO recipients [31]. Indeed, passive transfer of neutralizing anti-TNF mAb was unable to alter the mortality of the GKO CD4^+^ T cell recipients (data not shown). These results suggested additional factor(s) intrinsic to GKO CD4^+^ T cells in mediating disease outcome. Inhibition of IL-17 production by IFN-γ [37], suggested IL-17 as a potential candidate. Consistent with this concept, IL-17 mRNA expression was increased in the CNS of GKO CD4^+^ T cell recipients (Figure 3B), although IL-17 is not expressed in the CNS of infected WT mice [38]. Importantly, IL-17 mRNA remained below detection not only in SCID-infected control mice lacking T cells, but also in recipients of WT CD4^+^ T cells depleted of IFN-γ (Figure 3B), both of which are characterized by vast CNS neutrophil infiltration (Figure 1A). Although neutrophil-derived IL-17 has been implicated in enhancing tissue damage during reperfusion injury [39], these data suggest that neutrophils recruited into the CNS do not secrete IL-17 during acute viral encephalitis. Expression of IL-17 mRNA only in GKO CD4^+^ T cell recipients also ruled out a potential contribution of resident CNS cells. IL-17 expression exclusively in the CNS of GKO recipients thus implied that the source of IL-17 was the GKO-derived CD4^+^ T cell population itself. In support of this concept, transcript levels encoding IL-22, another cytokine produced by Th17 cells [40], were also significantly increased in infected GKO recipients compared to WT recipients and infected SCID control mice (Figure 3C). By contrast, IL-21, a CD4^+^ T cell-derived cytokine known to provide helper functions to CD8^+^ T cells and B cells [41] was expressed at similar levels in both the GKO and WT CD4^+^ T cell recipient groups (Figure 3C). IL-17 production by CD4^+^ T cells in the CNS of GKO recipients was confirmed by immunofluorescence histochemistry. A substantial fraction of T cells within the CNS of GKO recipients expressed IL-17. Moreover, all IL-17 positive cells co-expressed CD3 (Figure 3D), indicating that T cells are the predominant source of IL-17 within the CNS of SCID recipients. In contrast to the CNS, only ~8% of T cells in the cervical lymph nodes of GKO recipients secreted IL-17 at day eight p.i. (Figure 3E), suggesting enrichment of IL-17-expressing T cells within the CNS. To determine if IL-17 expression is imprinted during the primary response following immunization of GKO donor mice, cytokine expression was analyzed in the memory WT and GKO T cell populations prior to transfer (Figure 3F). WT memory CD4^+^ T cells prominently expressed IFN-γ and very little, if any, IL-17 following *in vitro* stimulation. By contrast, immunization of GKO mice primed a small fraction of memory CD4^+^ T cells capable of producing IL-17. These results were consistent with IFN-γ-mediated inhibition of Th17 cells [42] and suggested that IL-17 expression was imprinted prior to transfer and re-expressed in the infected recipients. To confirm a role of IL-17 in the early mortality of GKO recipients, WT and GKO recipients were treated with anti-IL-17 mAb. Consistent with the absence of IL-17 mRNA in the CNS of the WT recipients, anti-IL-17 treatment had no effect on the survival of WT recipients (Figure 4A). By contrast, inhibition of IL-17 in GKO recipients lead to a significant decrease in mortality, with 73% of mice surviving to day 18 p.i. (Figure 4A). In support of the concept that mortality was not influenced by neutrophils, the increased neutrophil infiltration in the CNS of GKO recipients was not altered by anti-IL-17 treatment (Figure 4B), confirming their primary regulation by IFN-γ [4].

### IFN-γ overcomes IL-17-derived CD4^+^ T cell mediating mortality

**Figure 5:**
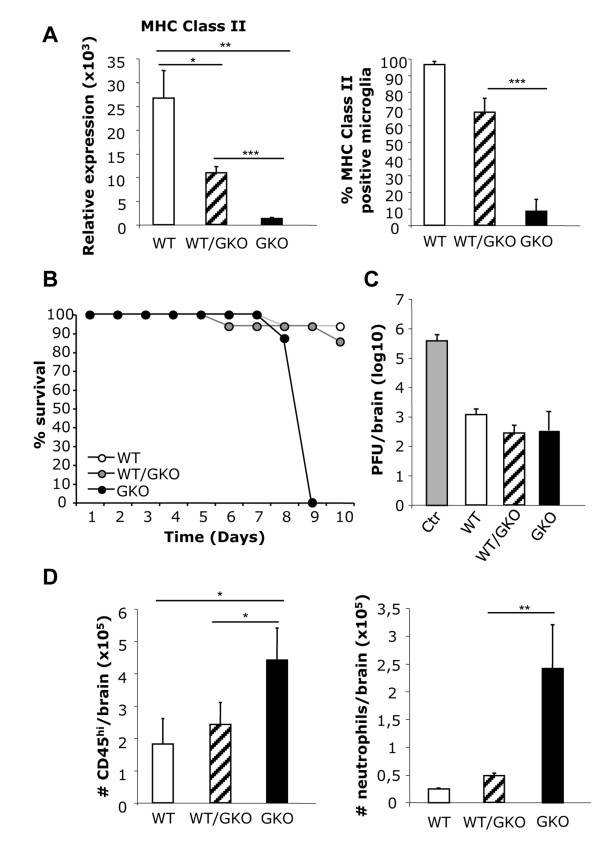
**WT CD4**^**+**^**T cell co-transfer prevents GKO CD4**^**+**^**T cell induced mortality. (A)** Relative MHC I-A^d^ class II mRNA expression in the CNS of infected SCID recipients of WT, WT/GKO or GKO CD4^+^ T cells determined by quantitative real-time PCR. Data represent the mean ± SEM of two independent experiments with n = 4 per experiment. Microglial MHC class II expression quantified by flow cytometry in infected SCID recipients of WT, WT/GKO or GKO CD4^+^ T cells. Data represent the mean ± SD of three separate experiments with n = 4 per group per experiment. **(B)** Survival was assessed following infection of SCID recipients of WT (n = 20), WT/GKO (n = 20) or GKO (n = 12) CD4^+^ T cells. Data represent four separate experiments. **(C)** Control of virus replication analyzed by plaque assay in infected control, SCID recipients of WT, WT/GKO or GKO CD4^+^ T cells at day eight p.i. Data represent the average (±SD) of eight mice per group combined from two independent experiments. **(D)** Total number of bone-marrow-derived leukocytes (CD45^hi^) and neutrophils (Ly6G^+^) analyzed by flow cytometry at day eight p.i. in brains of infected SCID recipients of WT, WT/GKO and GKO CD4^+^ T cells. Data represent the mean of twelve mice per group combined from three independent experiments.

Cross-regulation of IFN-γ and IL-17 in shaping CD4^+^ T cell subsets is well established during primary T cell activation and expansion [43,44]; however, less is known about cross-regulation during antigen-induced restimulation of memory T cells. IL-23 has recently been shown to promote GM-CSF expression by Th17 cells, which in turn enhances detrimental disease outcomes [27,28]. Based on the observation that GM-CSF is downregulated by IFN-γ [27], we tested whether IFN-γ-producing CD4^+^ T cells can override the detrimental function of GKO CD4^+^ T cell-mediated IL-17 expression. Memory CD4^+^ T cells from WT Thy1.1 immunized mice were co-transferred with Thy1.2 GKO CD4^+^ T donor cells (WT/GKO recipients) prior to infection. MHC class II expression was measured in the CNS of all recipient groups to confirm functional IFN-γ expression [34]. Class II mRNA was maximal in the CNS of WT recipients, reaching 22-fold higher levels than in GKO recipients. MHC class II mRNA was increased 10-fold following the co-transfer of WT and GKO CD4^+^ T cells compared to mice receiving GKO CD4^+^ T cells alone (Figure 5A). Increased mRNA expression correlated with MHC class II protein expression by the majority of microglia in both WT and co-transfer groups (Figure 5A). Although the proportion of microglia expressing MHC class II was decreased in the co-transfer compared to the WT groups, differences were not statistically significant (Figure 5A). Co-transfer of WT and GKO CD4^+^ T cells protected recipients from early mortality (Figure 5B). Disease severity was similar in WT and WT/GKO recipients and significantly reduced compared to GKO-only recipients, especially after day six p.i. (data not shown). In addition, control of CNS virus replication was identical at day eight p.i. in recipients of WT, WT/GKO or GKO CD4^+^ T cells (Figure 5C), confirming previous results that accelerated mortality does not correlate with uncontrolled virus replication [31]. CNS leukocyte infiltration in WT/GKO CD4^+^ T cell recipients was similar to WT recipients, and lower than GKO recipients (Figure 5D). Specifically, neutrophils within CD45^hi^ bone-marrow-derived inflammatory cells were reduced to 16% in WT/GKO recipients, resembling the proportion in WT recipients (Figure 5D and data not shown).

**Figure 6:**
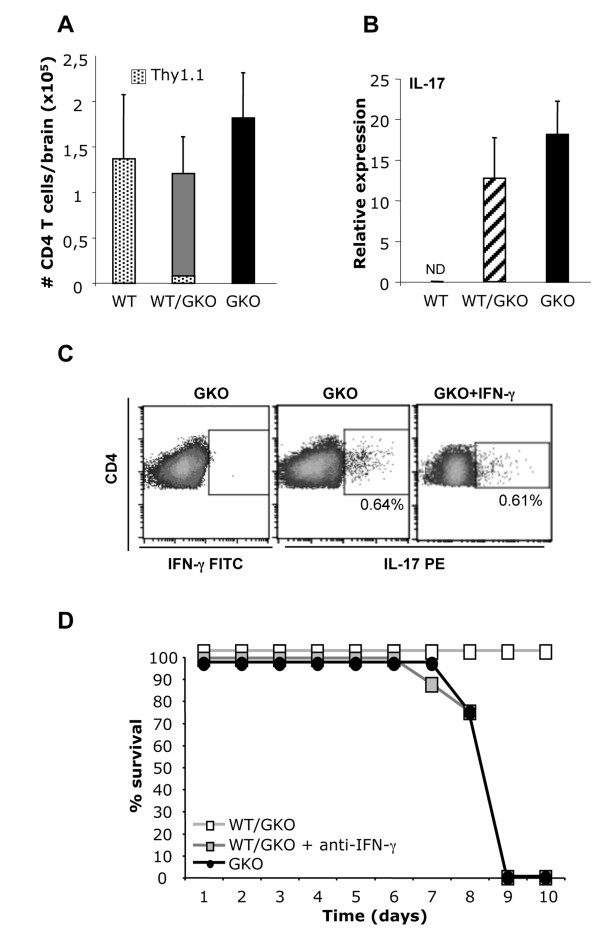
**IFN-γ-mediated protection prevents IL-17-mediated mortality. (A)** Number of CD4^+^ T cells in the infiltrating population and distribution of Thy1.1 positive cells measured by flow cytometry at day eight p.i. Data represent means (±SD) of twelve mice per group combined from three separate experiments. **(B)** IL-17 mRNA expression determined by quantitative real-time PCR in infected SCID recipients of WT, WT/GKO or GKO CD4^+^ T cells. Data represent the mean of two experiments with n = 4 in each group per experiment. **(C)** Splenocytes of immunized GKO donors cultured in the presence of JHMV with or without recombinant IFN-γ (10 ng/ml) for six days and restimulated four hours with PMA/Ionomycin. Intracellular cytokine expression on CD4^+^ T cells analyzed by flow cytometry using FITC-IFN-γ, PE-IL-17 and the corresponding isotype controls. Dot plots are representative of duplicates from two separate experiments. **(D)** Survival of infected SCID recipients of WT/GKO (n = 6), WT/GKO + anti-IFN-γ mAb (n = 8) and GKO (n = 4) CD4^+^ T cells assessed daily. Data are representative of two separate experiments.

Overall these data confirm IFN-γ-mediated control of CNS neutrophil infiltration and suggested a protective role of IFN-γ during viral encephalitis, via inhibiting IL-17 effector function by either directly reducing Th17 cell expansion and/or CNS entry, or limiting GM-CSF production. To assess whether GKO CD4^+^ T cells migrated to the CNS in the presence of WT CD4^+^ T cells, the relative proportions of each donor population was examined by co-transfer of Thy1.1 WT CD4^+^ T cells and Thy1.2 GKO CD4^+^ T cells. Surprisingly, T cells recruited into the CNS of infected co-transferred recipients were essentially derived from the GKO memory CD4^+^ T cells, as less than 20% of CD4^+^ T cells expressed Thy1.1^+^ (Figure 6A). Given the large population of infiltrating GKO CD4^+^ T cells, we next determined if IFN-γ-mediated protection correlated with reduced IL-17 mRNA expression. Although protective, the minor population of WT CD4^+^ T that infiltrated the CNS did not reduce expression of IL-17 mRNA in the CNS (Figure 6B). Protection mediated by IFN-γ, despite elevated IL-17, suggested that IFN-γ interferes with IL-17-mediated signaling events, rather than directly influencing Th17 expression. This notion was tested by *in vitro* stimulation of memory CD4^+^ T cells derived from GKO donors in the presence of recombinant IFN-γ. Exogenous IFN-γ was indeed unable to downregulate IL-17 production (Figure 6C), supporting the *in vivo* observation that IFN-γ-expressing WT CD4^+^ T cells did not alter CNS expression of IL-17 mRNA in WT/GKO recipients (Figure 6B). The maintenance of IL-17 in the presence of IFN-γ *in vitro* and *in vivo* indicates that the phenotypes acquired during *in vivo* primary responses are retained in the transferred memory cells following reactivation in recipient mice. To confirm this assumption, IFN-γ was depleted in WT/GKO recipients. WT/GKO recipients treated with anti-IFN-γ succumbed to infection by day nine p.i. similar to infected recipients of GKO CD4^+^ T cell (Figure 6D). These data actually suggest that IFN-γ diminishes the detrimental effects of IL-17, despite the apparent expansion/survival advantage of GKO relative to WT CD4^+^ T cells in the infected recipients.

**Figure 7:**
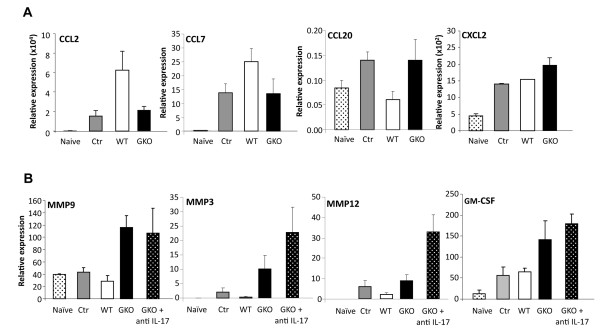
**Alterations in chemokines, MMPs and GM-CSF mRNA do not correlate with IL-17-mediated mortality. (A)** mRNA expression analyzed in the CNS of naïve mice, infected control mice, and SCID recipients of WT or GKO CD4^+^ T cells at day eight p.i. Data represent the mean (±SEM) of four individual mice per group **(B)** MMP9, MMP3, MMP12 and GM-CSF mRNA expression measured in the CNS of naïve, controls, infected SCID recipients of WT, GKO or GKO + anti-IL-17 CD4^+^ T cells at day eight p.i.. Data are representative of the mean (±SEM) of four individual mice per group.

To determine potential mechanisms of IL-17-mediated mortality, IL-17-dependent chemokines and matrix metalloproteinases (MMPs) [45] were analyzed in JHMV-infected SCID recipients after transfer of WT or GKO CD4^+^ T cells. Similar expression of CCL2, CCL7 and CCL20 was detected comparing infected SCID controls and GKO recipients; by contrast CCL2 and CCL7 were upregulated and CCL20 downregulated in recipients of WT CD4^+^ T cells (Figure 7A). These data suggest that in contrast to EAE, CCL2, CCL7 and CCL20 chemokine expression is regulated by IFN-γ rather that IL-17 during JHMV infection. Moreover, no significant difference in CXCL2 mRNA was found comparing SCID-infected controls and recipients of either WT or GKO CD4^+^ T cells (Figure 7A), supporting CXCL1 as the major neutrophil chemoattractant during JHMV infection. CNS infection with JHMV induces a limited number of MMPs, that is, MMP9, MMP3 and MMP12 [46]. As MMP9 is specifically expressed by neutrophils [47], abundant neutrophil recruitment in the CNS of GKO T cell recipients (whether or not treated with anti-IL17) correlated with MMP9 expression (Figure 7B). MMP3 and MMP12 mRNA expression were also upregulated in GKO recipients compared to infected SCID controls and WT recipients, suggesting a potential role of these MMPs in GKO mortality by mediating tissue destruction (Figure 7B). However, survival of GKO recipients treated with anti-IL17 also expressed increased MMP3 and MMP12 mRNA (Figure 7B), suggesting that MMP3 and MMP12 play no role in the early mortality of GKO recipients. Finally, to investigate a potential contribution of GM-CSF to the rapid disease progression, relative levels of GM-CSF were measured in the CNS of SCID-infected controls, and recipients of WT and GKO CD4^+^ T cells. GM-CSF mRNA expression was increased in GKO recipients relative to controls and WT CD4^+^ T cell recipients. These data were reminiscent of enhanced GM-CSF expression by Th17 compared to Th1 cells in EAE [27] and suggested a potentially detrimental role during JHMV encephalomyelitis. However, the increased survival of GKO recipients treated with anti-IL17 mAb did not correlate with a decrease in GM-CSF expression. These results indicate that GM-CSF expression correlated with IFN-γ deficiency, but not with an IL-17 mediated feedback loop. Nevertheless, these data suggest that IFN-γ directly affords protection from mortality by interfering with detrimental IL-17-mediated events, distinct from those mediating EAE.

## Discussion

IFN-γ and IL-17 are major effector molecules of tissue inflammation that play opposing roles in neutrophil recruitment/accumulation [4,48,49]. While their distinct influence on disease has been demonstrated during autoimmune-mediated neuroinflammatory responses, the interplay between IL-17 and IFN-γ, specifically the effects on downstream targets remain controversial. Furthermore, during microbial infections, protective and detrimental effects of IFN-γ and IL-17 depend on the pathogen and prominent cell types affecting microbial control [50-52]. The present study evaluated how the absence of IFN-γ secretion by CD4^+^ T cells contributes to a rapid lethal outcome during viral encephalomyelitis, without altering viral control. Early virus-induced mortality in SCID recipients of GKO virus-specific memory CD4^+^ T cells correlated with both IL-17 production and extensive neutrophil accumulation in the CNS. Selective blockade of either neutrophils or IL-17 demonstrated that early mortality did not correlate with CNS neutrophil recruitment, but rather with IL-17. This was confirmed by the prolonged survival of recipients of anti-IFN-γ mAb-treated WT recipients, which were characterized by extensive neutrophil inflammation, but an absence of IL-17.

Neutrophil-independent pathogenic effects of IL-17 in the JHMV model contrast with non-CNS viral infectious models including the influenza virus and herpes simplex virus-1 infections, which attribute Th17 cell-mediated pathogenesis to neutrophil attraction [12,14]. However, neutrophil depletion following severe influenza virus infection also suggests that neutrophils play a protective, rather than a deleterious role [53]. Our data also contrast with the deleterious role of neutrophils during EAE [49,54]. Adoptive transfer of Th17 cells leads to excessive CNS neutrophil migration after EAE induction, while impaired neutrophil recruitment restrains leukocyte access into the CNS [49], indicating a prominent role of neutrophils in disrupting the blood-brain barrier. However, in contrast to EAE, neutrophils are not essential for the loss of blood-brain barrier integrity following sublethal JHMV infection [55]. By contrast, JHMV-induced encephalomyelitis demonstrates that IFN-γ plays a more prominent role than IL-17 in regulating CNS neutrophil recruitment and/or retention by downregulating ELR^+^ neutrophil chemokine expression. Increased neutrophils correlated with high CXCL1 expression in the CNS of both IFN-γ-depleted WT recipients lacking IL-17, as well as in GKO recipients treated with anti-IL-17 Ab. Moreover, neutrophil infiltration was reduced by co-transfer of WT and GKO CD4^+^ T cells, despite sustained IL-17 expression in the CNS. These results are consistent with early studies identifying IFN-γ as a critical factor regulating CNS neutrophil infiltration [4], as well as recent observations implicating IFN-γ as a dominant molecule controlling CNS inflammation [26].

Despite evidence implicating IL-17 as a pathogenic mediator, independent of neutrophils, the mechanism(s) involved in IL-17-induced mortality of JHMV-infected mice remain unclear. Identical viral burden at day eight p.i. in all recipients [31] indicated that IL-17 does not alter control of virus replication, in contrast to its role in facilitating viral persistence following Theiler’s murine encephalomyelitis virus infection [15]. Sustained Ag independent interaction between Th17 and neuronal cells during EAE correlated with increased neuronal damage due to IL-17-mediated neurotoxicity [25]. Increased gray matter infection, especially in neuronal cells, is associated with premature death following JHMV infection of mice deficient in innate immune components [56]. In addition, there is a preferential distribution of CD4^+^ T cells in the gray matter of GKO recipients compared to WT recipients [31], suggesting the possibility that in absence of IFN-γ, IL-17-secreting CD4^+^ T cells localize proximal to uninfected neurons, contributing to neuronal dysfunction and premature death. However, few neurons are infected early during JHMV pathogenesis in SCID mice and the types of infected cells were similar in all groups, suggesting no alteration in viral tropism [31]. In addition, no differential neuronal loss was found comparing GKO and WT recipients [31]. Similarly, increased expression of GM-CSF in GKO recipients compared to the WT counterparts suggested that GM-CSF might also contribute to disease outcome following JHMV infection. GM-CSF was implicated as a pathogenic effector molecule secreted by Th17 cells during EAE [27,28]. However, the survival of GKO recipients treated with anti-IL17 did not correlate with a decrease in GM-CSF expression. Although GM-CSF expression is reduced by IFN-γ [27], the data do not support a pathogenic role of GM-CSF in early mortality of JHMV-infected GKO recipients.

IL-17 mRNA expression in GKO CD4^+^ T cell recipients suggested Th17 cells as the primary mediators of disease. Nevertheless, IL-17 can also be produced by neutrophils, γδ T cells, NK and CD8^+^ T cells [57,58]. A deleterious contribution of neutrophil-derived IL-17, suggested during kidney ischemia-reperfusion [39], was ruled out by the inability of neutrophil-depletion to rescue mice from early death, as well as the absence of IL-17 mRNA in WT recipients treated with anti-IFN-γ, despite high CNS neutrophil infiltration. IL-17 production by CD4^+^ T cells derived from immunized GKO donors prior to transfer supports GKO CD4^+^ T cells as the primary source of IL-17. Moreover, stimulation of WT donor CD4^+^ T cells strongly induced IFN-γ, but not IL-17, indicating that virus-specific Th17 cells only differentiate in the absence of IFN-γ. These results support previous observations of a minor, if any, role of Th17 cells in the pathogenesis of JHMV-infected immunocompetent WT mice [38] and corroborate the inhibitory function of IFN-γ on Th17 differentiation during T cell priming [59]. However, our data are novel in demonstrating that memory GKO CD4^+^ T cells are committed in their ability to produce IL-17 when restimulated in the recipient host, even in the presence of IFN-γ. Although unanticipated, this finding was confirmed by the inability of IFN-γ to downregulate IL-17 production in GKO donor cells *in vitro*, as well as on *in vitro*-differentiated mature Th17 cells [15]. Similarly, the IL-27 suppressive function on Th17 differentiation from naïve CD4^+^ T cells could not be reproduced on memory Th17 cells [60], supporting the stability of committed Th17 cells. Importantly, the prolonged survival of co-transferred recipients, despite sustained CNS IL-17 expression, suggests that IFN-γ overcomes the deleterious effects of IL-17. However, the mechanisms by which IFN-γ overrides IL-17 function remain unclear. In EAE, IL-17 exerts detrimental effects via signaling in resident CNS cells, with astrocytes implicated as major targets [45]. However, Th17 cell localization proximal to neurons also implicates potential dysregulation of neuronal function [25]. Responsiveness of both cell types to IFN-γ [61,62] suggests IFN-γ may counteract signaling molecules downstream of the IL-17R.

## Conclusions

This study demonstrates that IL-17, in the absence of IFN-γ, can accelerate mortality during viral encephalomyelitis by a mechanism independent of the magnitude of CNS neutrophil infiltration and reversible by IFN-γ.

## Abbreviations

BSA, bovine serum albumin; CNS, central nervous system; DAPI, 4’-6-diamidino-2-phenylindole; EAE, experimental autoimmune encephalitis; FITC, fluorescein isothiocyanate; GKO, IFN-γ deficient; GM-CSF, granulocyte monocyte colony-stimulating factor; H&E, hematoxylin and eosin; IFN-γ, interferon-gamma; IL, interleukin; i.p., intraperitoneal; i.v., intravenous; JHMV, gliatropic JHM strain of mouse hepatitis virus; mAb, monoclonal antibody; MHC, major histocompatibility complex; MMP, matrix metalloproteinase; PCR, polymerase chain reaction; p.i., post infection; PE, phycoerythrin; PerCP, peridinin chlorophyll protein; SCID, severe combined immunodeficiency; SD, standard definition; SEM, standard error of the mean; TNF, tumor necrosis factor; WT, wild-type.

## Competing interests

The authors declare they have no competing interests.

## Authors’ contributions

CS designed and performed the experiment, collected and analyzed data, and wrote the manuscript. SAS designed and performed the research, interpreted data and wrote the manuscript. DRH analyzed and interpreted data. RMR interpreted data. DJC provided materials, interpreted data and edited the manuscript. CCB designed the research, provided materials, interpreted data and wrote the manuscript. All authors read and approved the final manuscript.
